# Adaptive Treatment Strategy: Adjuvant Chemoradiotherapy for a Complex SMARCB1 (INI-1)-Deficient Sinonasal Carcinoma With Intracranial Extension

**DOI:** 10.7759/cureus.77217

**Published:** 2025-01-10

**Authors:** Mansoor Qayoumi, Ayesha Mushtaq, Michael Jansen, Seamus O'Reilly, Muhammad Jamaluddin

**Affiliations:** 1 Internal Medicine, Cork University Hospital, Cork, IRL; 2 Radiation Oncology, Cork University Hospital, Cork, IRL; 3 Neuropathology, Cork University Hospital, Cork, IRL; 4 Medical Oncology, Cork University Hospital, Cork, IRL

**Keywords:** concomitant chemoradiation therapy, palliative radiation therapy, proton beam radiotherapy, vmat radiotherapy, volumetric‐modulated arc therapy (vmat)

## Abstract

SWI/SNF‐related matrix‐associated actin‐dependent regulator of chromatin subfamily B member 1 (SMARCB1) (integrase interactor 1)-deficient sinonasal carcinoma (SDSNC) is a rare and aggressive malignancy of the head and neck. It is characterised by the absence of nuclear SMARCB1 expression and typically presents at advanced stages due to non-specific symptoms resembling benign conditions such as nasal polyps or sinusitis. This report describes a 64-year-old male who presented with a sphenoidal sinus mass causing bony erosion and intracranial extension, associated with headaches and diplopia. Initial management included endoscopic debulking surgery, followed by chemoradiotherapy to address residual disease in proximity to the optic chiasm and brainstem. Urgent radiotherapy was delivered using volumetric-modulated arc therapy, with a total dose of 66 Gy, requiring precise sparing of critical structures. Immunohistochemical analysis confirmed SMARCB1 deficiency. Post-treatment imaging revealed significant tumour volume reduction, and immune checkpoint inhibitor therapy was initiated for disease control. At five months post-treatment, the tumour remained stable.

This case highlights the diagnostic and therapeutic challenges of SDSNC, particularly in cases involving critical anatomic structures. It demonstrates the importance of multidisciplinary treatment strategies, advanced radiotherapy planning, and immunotherapy in optimising outcomes for this rare malignancy.

## Introduction

SWI/SNF‐related matrix‐associated actin‐dependent regulator of chromatin subfamily B member 1 (SMARCB1) (integrase interactor 1 (INI-1))-deficient sinonasal carcinoma (SDSNC) is a rare and aggressive malignancy predominantly affecting the nasal cavity and paranasal sinuses, accounting for fewer than 1% of all head and neck neoplasms [1]. The absence of nuclear SMARCB1 (INI-1) expression, identified through immunohistochemistry or cytogenetic abnormalities on chromosome 22q11.2, characterises this disease [2]. SDSNC typically presents at locoregionally advanced stages as the diagnosis is often delayed due to non-specific symptoms resembling benign conditions such as allergic rhinitis, nasal polyps, chronic sinusitis, or other malignancies such as human papillomavirus-associated squamous cell carcinoma, extranodal natural killer/T-cell lymphoma, and mucosal melanoma. For this reason, achieving complete surgical resection is frequently challenging, with most cases experiencing incomplete removal [1].

The recognition of SMARCB1-deficient sinonasal carcinoma (SDSNC) as a distinct entity emerged recently, with its initial description in 2014 [2,3]. Fewer than 200 cases of this type of sinonasal carcinoma have since been reported in the English-language literature, as indicated by a search conducted on PubMed. In this article, we present a complex case of SDSNC treated with an adaptive strategy involving surgery and adjuvant chemotherapy and radiotherapy. This is the first article to describe the radiotherapy planning challenges in SDSNC.

## Case presentation

A 64-year-old man with a history of well-managed epilepsy presented with a three-month history of worsening right-sided headache, accompanied by a two-week history of diplopia, nausea, and vomiting. Neurological examination revealed right-sided sixth nerve palsy. A CT and MRI of the brain identified a 5-cm isodense mass occupying the right sphenoidal sinus with surrounding bony erosions and extension into the right temporal region.

**Figure 1:**
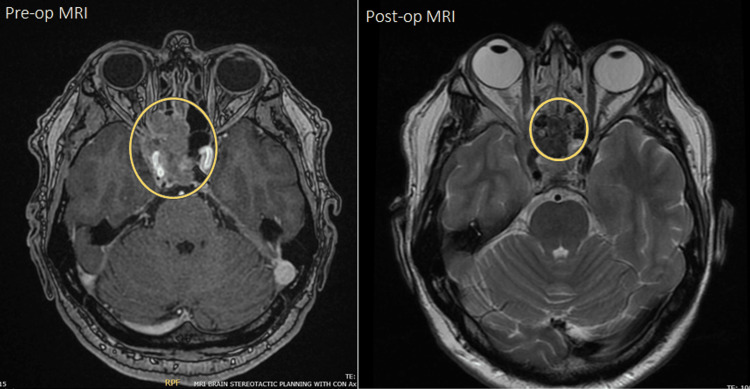
Preoperative and postoperative MRI of the brain.

After a neurosurgical consult, urgent endoscopic debulking was performed. The patient underwent endoscopic trans-sphenoidal trans-ethmoidal surgery to debulk a midline skull base tumour involving the sphenoid sinus, clivus, right cavernous sinus, sella, and superior orbital fissure. Postoperatively, his sixth nerve palsy resolved, and he was discharged three days later. Postoperative scans revealed a residual mass extending into the right cavernous sinus and the right middle cranial fossa, slightly displacing the right temporal lobe (Figure 1).

**Figure 2:**
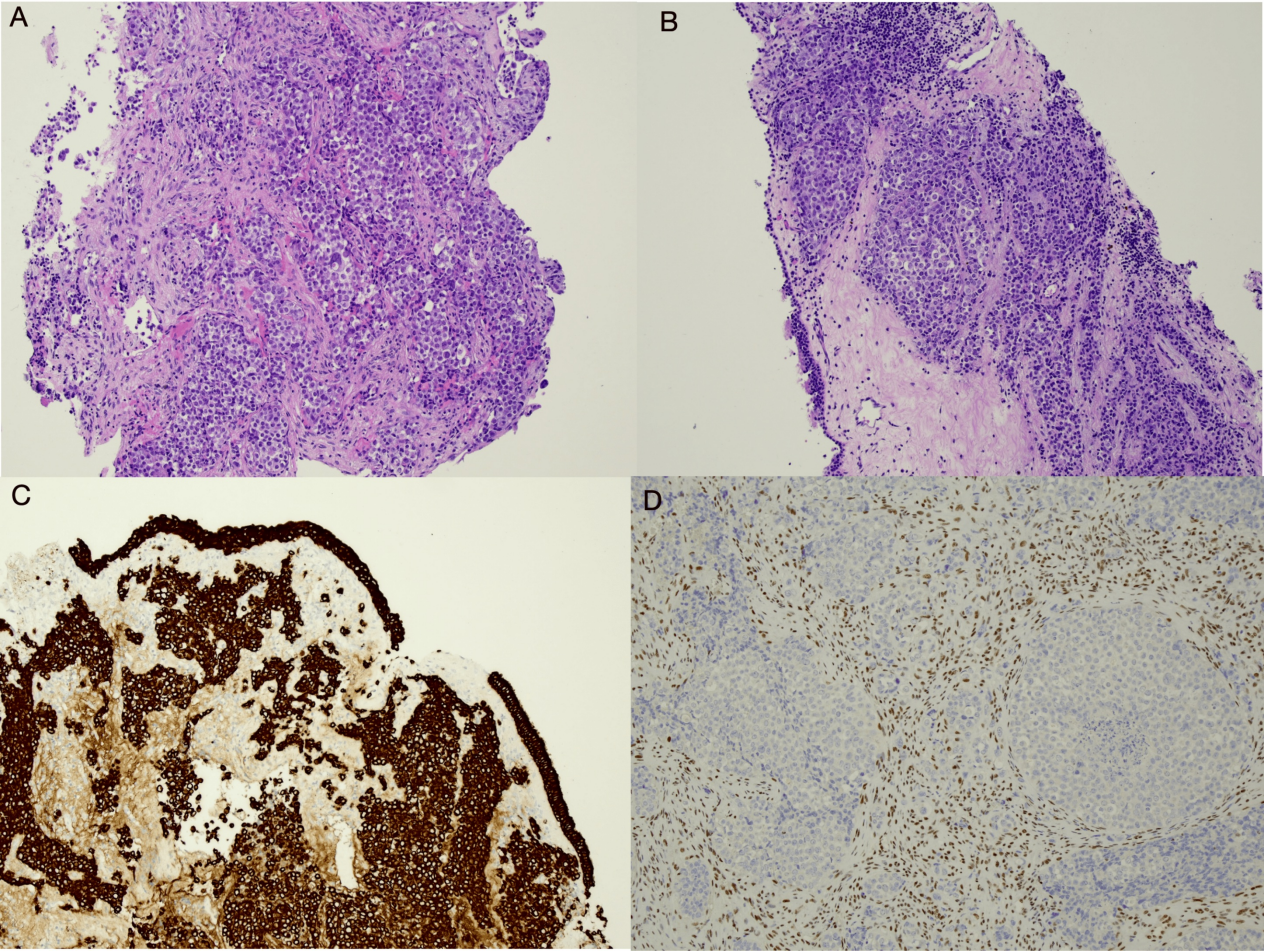
Histopathologic and immunohistochemical slides of the patient’s tumour. (A, B) HE stain: the tumour consists of sheets of undifferentiated cells undermining included respiratory epithelium. (C) MNF-116 pan-keratin immunohistochemistry: strongly labels cells in keeping with carcinoma. (D) INI-1 immunohistochemistry: loss of signal indicating SWI/SNF inactivation in tumour cells, with the surrounding desmoplastic stroma showing retained signal in cell nuclei (internal control). All images: HE and immunohistochemistry magnification (40×). HE: hematoxylin and eosin; MNF-116: cytokeratin pan monoclonal antibody; SWI/SNF: SWItch/sucrose non-fermentable; INI-1: integrase interactor 1

Histopathological and immunohistochemical examination of the tumour revealed an ethmoid sinus locally invasive mass, poorly differentiated carcinoma and INI-1 (SMARCB1) negative (Figure 2).

Following multidisciplinary discussion, a decision was made that the residual disease could be treated with carboplatin (AUC 5) and 5-fluorouracil 1,000 mg/m^2^/day (28-day cycle) followed by radiation. Due to the proximity of the disease to critical structures such as the optic chiasm and brainstem, it was suggested that particle therapy would be more appropriate and referral to a third outside institution was sought.

**Figure 3:**
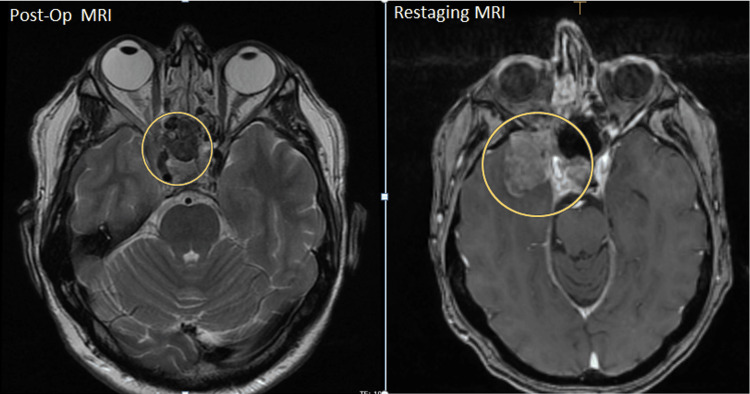
Re-staging MRI compared with postoperative imaging.

After the first cycle of chemotherapy, the patient experienced a generalized tonic-clonic seizure along with right facial asymmetry and decreased right visual acuity. MRI of the brain and sinuses revealed rapid interval growth of the residual lesion with new extension into the right temporal lobe, the apex of the right retro-orbital region, and through the posterior wall of the right maxillary sinus (Figure 3).

Due to the urgency of the clinical situation, the patient had to commence radiotherapy on an urgent basis using photons, with the hope to complement with particle therapy to be able to escalate the dose to the tumour and maximize organs-at-risk (OAR) sparing. The particle treatment centre was consulted after the third fraction of photon treatment, and it was felt for safe practice to complete the treatment in the same institution using just photons rather than a combination of external-beam radiotherapy and particle therapy.

Following an urgent CT simulation scan, treatment volumes were delineated, and the patient began radiotherapy using volumetric-modulated arc therapy, receiving 60 Gy in 30 fractions. Due to the tumour’s location, the right optic nerve was sacrificed to achieve tumour control. The primary OAR identified through MRI fusion included the brainstem (Dmax: 53.9 Gy), optic chiasm (Dmax: 53.92 Gy), left optic nerve (Dmax: 53.65 Gy), and left lens (Dmax: 5.84 Gy).

**Figure 4:**
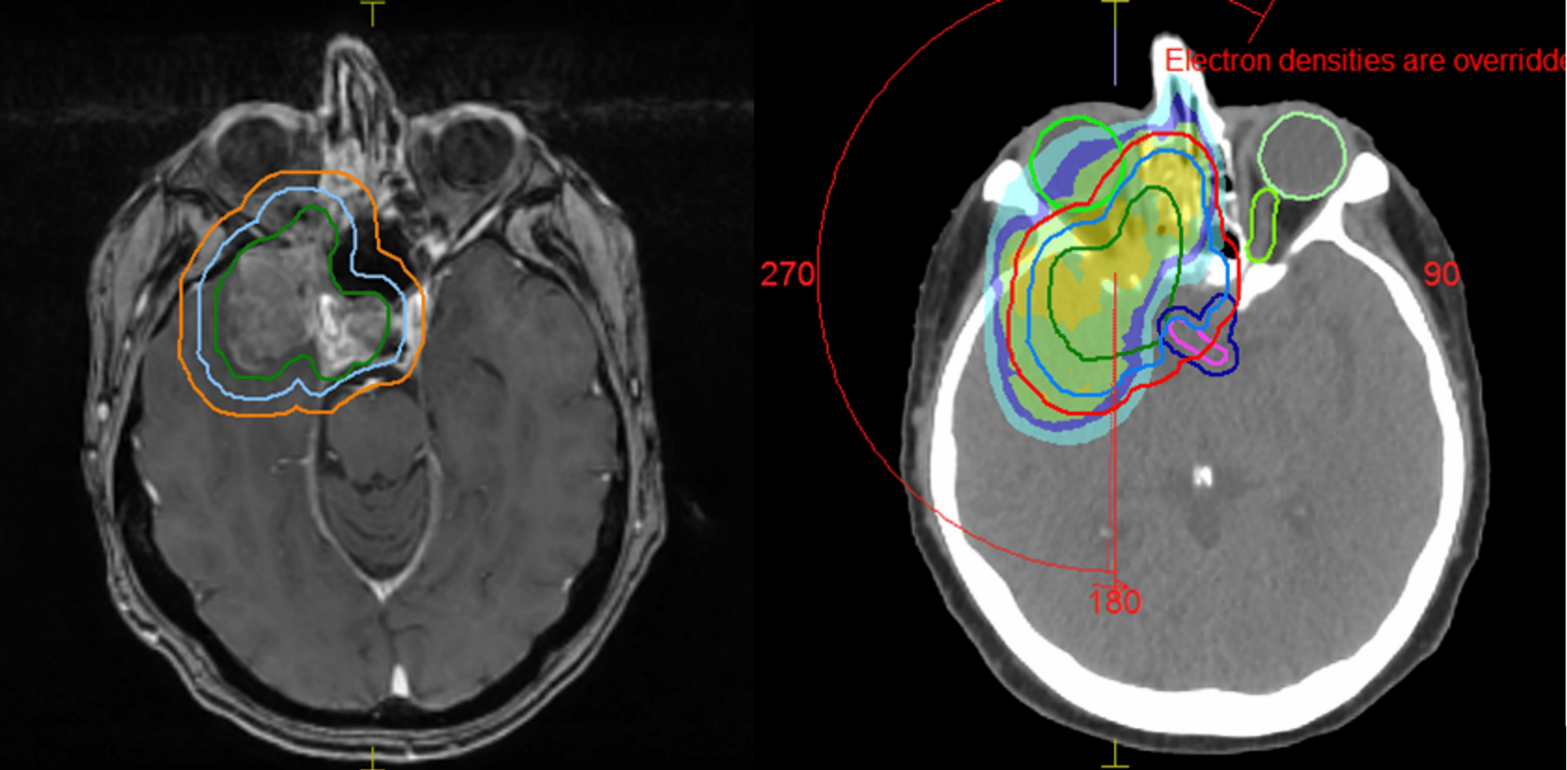
Planning scan. GTV (green), CTV (blue), and PTV (orange). Sparing of the left optic nerve and optic chiasm. GTV: gross tumour volume; CTV: clinical target volume; PTV: planning target volume

After 20 fractions (40 Gy), an interim MRI was requested to evaluate tumour response which revealed a reduction in the gross tumour volume. Repeat CT scan and planning ensued, and the dose was escalated to 66 Gy (additional 26 Gy in 13 fractions) without compromising the dose to the optic chiasm and brainstem (Figure 4).

**Figure 5:**
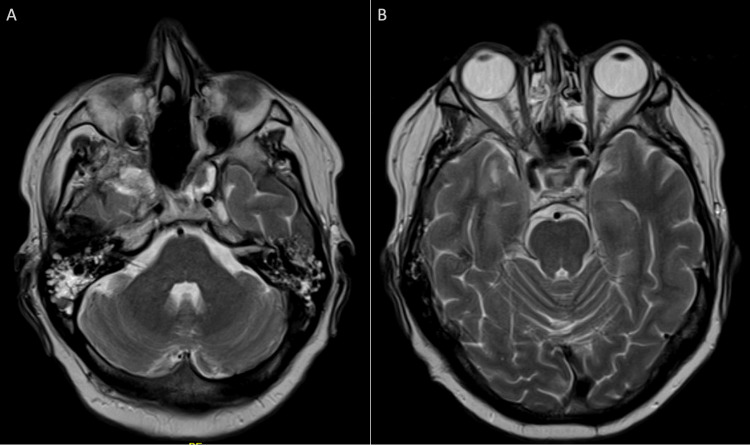
MRI of the brain with contrast (four weeks post-radiothrapy completion). Axial T2-weighted MRI images (A) at the level of the posterior cranial fossa and (B) at the level of sella turcica. MRI of the brain with contrast performed four weeks after completion of radiotherapy shows a marked reduction in tumour size following treatment.

The patient tolerated the treatment well, experiencing only mild skin irritation (Radiation Therapy Oncology Group grade 1) and dry eyes. Follow-up MRI of the brain and sinuses four weeks post-radiotherapy revealed a marked reduction in tumour volume, with residual volume involving the cavernous sinus and pituitary fossa, extending into the middle cranial fossa, and along the sphenoid wing (Figure 5).

**Figure 6:**
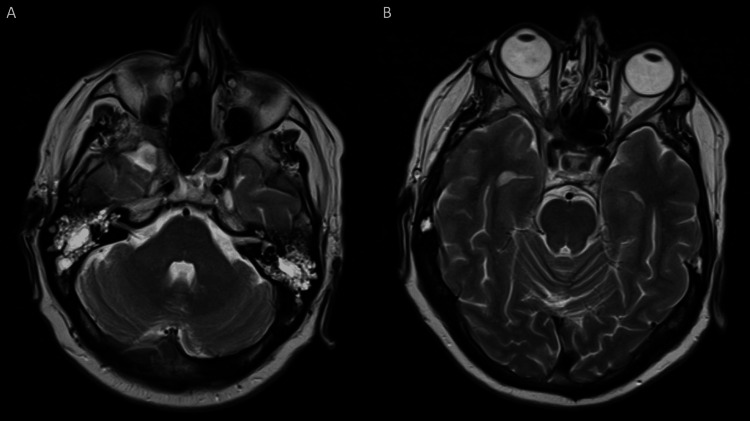
Follow-up MRI of the brain with contrast. Axial T2-weighted MRI images (A) at the level of the posterior cranial fossa and (B) at the level of sella turcica. MRI of the brain with contrast performed four months after the initiation of adjuvant chemotherapy demonstrates further reduction in the size of the residual tumour compared to the MRI obtained four months earlier.

Genetic testing with Oncomine found no actionable mutations, but programmed death-ligand 1 testing was positive. Hence, the patient was started on nivolumab (480 mg monotherapy administered every four weeks) to which he had a great response (Figure 6). As of the last follow-up, five months post-radiotherapy, the sinonasal tumour remained stable. The patient continues to be monitored regularly by the oncology team at our cancer institute.

## Discussion

Sinonasal carcinomas account for approximately 3% of head and neck neoplasms and often present at locally advanced stages, posing significant treatment challenges [4]. SDSNC, first identified in 2014, remains a rare and poorly understood malignancy of the sinonasal tract accounting for fewer than 1% of head and neck malignancies [5]. Historically, treatment strategies for sinonasal carcinomas did not differentiate based on specific subtypes, generally involving a combination of surgery, chemotherapy, and radiotherapy [6].

SMARCB1 (also known as INI-1), a core subunit of the SWI/SNF chromatin remodelling complex, functions as a tumour suppressor. Its loss rapidly induces cancer in experimental models [7]. The gene products of SMARCB1 are expressed in the nuclei of all normal human cells. Biallelic inactivation of SMARCB1 leads to a complete loss of expression, detectable via immunohistochemical staining, the standard diagnostic criterion for SMARCB1-deficient malignancies [8].

Differential diagnoses for SDSNC include olfactory neuroblastoma, sinonasal undifferentiated carcinoma, and non-keratinizing squamous cell carcinoma. Additionally, newly described entities such as NUT-midline carcinoma must be considered. Some tumours initially classified under these categories have shown loss of INI-1, with many displaying basaloid morphology and partial rhabdoid differentiation [6].

Patients with SDSNC frequently present with large, locally advanced tumours, often staged as T4 at diagnosis. The high morbidity associated with surgical resection suggests the potential benefit of neoadjuvant chemoradiation to reduce tumour volume before surgery. However, many patients historically underwent surgery followed by adjuvant chemoradiation with mixed outcomes [5,9].

Current treatment strategies typically involve surgery with adjuvant chemotherapy and/or radiotherapy. Charged-particle therapy, including proton and carbon ion therapy, has also been considered to spare critical structures such as the optic nerves and chiasm from radiation-induced damage and has shown promise in achieving high local and regional control rates with minimal severe toxicities [10,11]. Induction chemotherapy with TPF (docetaxel, cisplatin, and 5-fluorouracil) followed by radical chemoradiation is another approach, particularly for inoperable tumours [1]. Novel therapies, including immune checkpoint inhibitors, are under investigation, although their efficacy for SMARCB1-deficient carcinoma remains uncertain [12].

The prognosis for SDSNC is generally worse compared to its SMARCB1-retained carcinomas, particularly in advanced T4a/T4b disease. The largest systematic review of SDSNSCC found that one-year, two-year, and three-year overall survival (OS) rates were 84.3%, 62.9%, and 51.8%, respectively, with a median OS of 39.0 months. Male patients and those with T4b disease had significantly shorter OS compared to their counterparts [1].

This case uniquely addresses adaptive treatment strategy in the treatment of SDSNC, with an emphasis on radiotherapy planning challenges, especially given the tumour’s proximity to critical structures such as the optic chiasm and its potential extension into the middle cranial fossa and temporal lobe. These factors complicate treatment and necessitate precise and innovative radiotherapy techniques to maximise efficacy while minimising adverse effects.

## Conclusions

SDSNC remains a rare and aggressive malignancy, presenting significant treatment challenges due to its anatomical complexity and late diagnosis. Our case highlights the need for an adaptive approach combining surgery, chemotherapy, and radiotherapy to address the tumour’s proximity to critical structures. The decision to use photon therapy in our case, after considering particle therapy, underscores the importance of flexibility in treatment planning.

Future advancements in targeted therapies and immune checkpoint inhibitors, along with the utilisation of improved radiotherapy techniques, are essential for better outcomes. A multidisciplinary approach is crucial, as it can help address the unique challenges posed by this rare carcinoma and improve both survival and quality of life for patients.
